# Emergency surgical decompression and long-term survival of glioblastoma presenting in coma in old age: case report and review of prognostic factors

**DOI:** 10.1093/omcr/omac074

**Published:** 2022-07-26

**Authors:** Aidan McKinley, Simon Lammy, Allan James, Alexandru Stan, Chris Barrett

**Affiliations:** Department of Neurosurgery, Institute of Neurological Sciences, Queen Elizabeth University Hospital, Glasgow, UK; Department of Neurosurgery, Institute of Neurological Sciences, Queen Elizabeth University Hospital, Glasgow, UK; University of Glasgow, Glasgow, UK; Woodson Wohl Cancer Research Centre, Institute of Cancer Sciences, Garscube Estate, Glasgow, UK; Department of Clinical Oncology, Beatson West of Scotland Cancer Centre, Glasgow, UK; Laboratory Medicine Building, Queen Elizabeth University Hospital, Glasgow, UK; Department of Neurosurgery, Institute of Neurological Sciences, Queen Elizabeth University Hospital, Glasgow, UK

## Abstract

We present a case demonstrating that older age does not exclude long-term survival with glioblastoma. This is a malignant neoplasm with a median life expectancy of 14 months in patients treated with radical intent. Survival is dependent on several independent and interacting prognostic factors of which advancing age is a negative factor. We present a septuagenarian with a 3.5-year survival following aggressive management. The potential to improve glioblastoma survival in an elderly population by examination of additional prognostic factors and identifying biomarkers warrants further research.

## INTRODUCTION

Glioblastoma is the most common malignant primary brain tumour (~20%). Median survival is ~14 months in those treated radically: surgery followed by 60 Gy radiotherapy (XRT) and concomitant and 6-month sequential Temozolomide (TMZ). This standard of care was limited to patients < 70 years due to concerns regarding prognosis from advancing age and reduced tolerance to oncological therapy. Despite several trials addressing aggressive treatment in this older population (> 65 years), median survival remain short, i.e. 4 months for best supportive care to 9 months from combined chemotherapy and short course (40 Gy) XRT [1, 2]. We present the case of a septuagenarian countering such odds to become a long-term glioblastoma survivor for 3.5 years (whilst being IDH-1 wildtype).

## CASE SUMMARY

**Figure 1:**
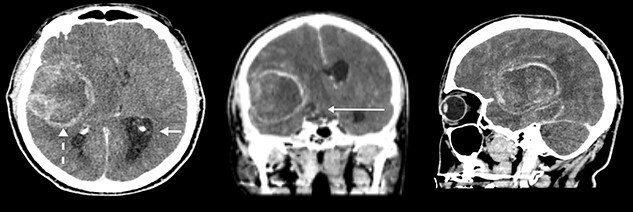
(**A**–**C**) Contrast CT at presentation (axial, coronal and sagittal) demonstrating a large right temporal lesion and peri-lesional oedema (dashed arrow), contralateral hydrocephalus (short straight arrow), causing significant mass effect, including uncal and subfalcine herniation (long straight arrow).

A 70-year-old man presented to hospital in June 2014. He had mild confusion, 24 h of left leg weakness and 6 months of headache. He was an ex-smoker of 43 years and enjoyed regular exercise. His baseline Karnofsky Performance Scale (KPS) score was > 90. A contrast CT (Fig. 1**A**–**C**) demonstrated two lesions: 65 × 45 × 43 mm (103 378 mm^3^) irregularly contrast enhancing mass in the right temporal lobe and a smaller 25 × 20 × 08 mm (7853 mm^3^) mass inferiorly to it. Total volume was 111 231 mm^3^. This caused 12 mm midline shift, hydrocephalus and midbrain compression. High-dose dexamethasone was commenced.

**Figure 2:**
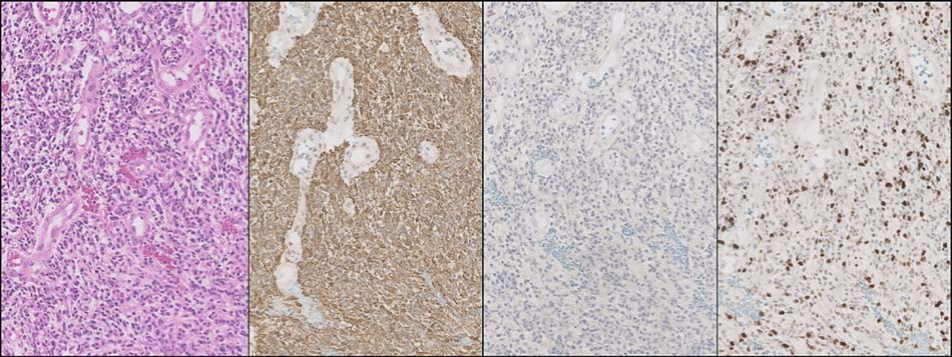
Histology and immunohistochemistry demonstrating pleomorphic cells and nuclear atypia (**A**), glial fibrillary acidic protein (GFAP) expression (**B**), IDH-1^wt^ (**C**) and Ki67 proliferation (**D**); from left to right. Original magnification ×200.

**Figure 3:**
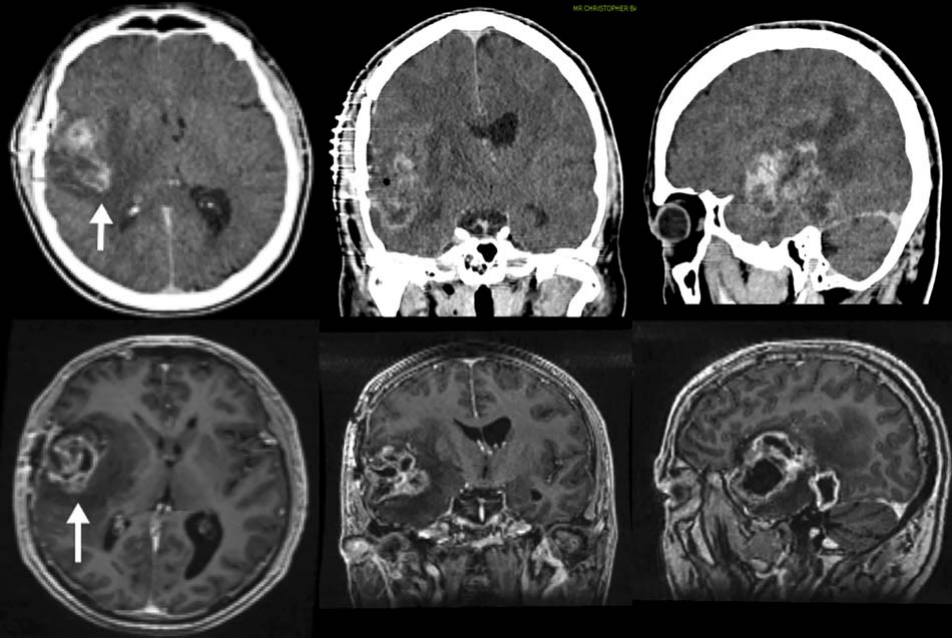
(**A**–**C**) (Top Row: axial, coronal and sagittal) contrast CT conducted < 72 h post-operatively demonstrating significant lesional reduction and mass effect (arrow). (Bottom row: axial, coronal and sagittal) Contrast T1W MRI demonstrating multiple areas of lesional enhancement representing a STR but a slight reduction in oedema (arrow). Patient could not tolerate the usual post-operative MRI due to poor neurological status. This was conducted at 14 days.

On transfer to our unit 9 h later, he deteriorated from GCS 15 to GCS 7 (localizing to pain, no eye opening and no verbal response). A repeat CT revealed worsening mass effect. 1 g of Levetiracetam (LEV) was given (and continued twice daily) orally and an overnight emergency surgical resection was performed to save life. The patient took several days to recover. Histopathology revealed a glioblastoma (WHO grade IV) (Fig. 2**A**–**D**). Molecular markers revealed isocitrate dehydrogenase 1 (IDH-1) wildtype and O^6^-methylguanine-DNA methyltransferase (MGMT) methylation. Ki67-index was ~ 25%. A post-operative CT and MRI (Fig. 3**A**–**C**) demonstrated a 95% subtotal resection (STR), as residual enhancement persisted, making a post-operative volume of 24 × 14 mm (5277 mm^3^). He underwent radical radiotherapy (XRT) of 60GY in 30 fractions and concomitant TMZ in September 2014. This was followed by 6 months of TMZ finishing in February 2015. Radiological surveillance was 3-monthly. The patient had a good response and his KPS score was < 70%. He was independent at home in all activities of daily living.

**Figure 4:**
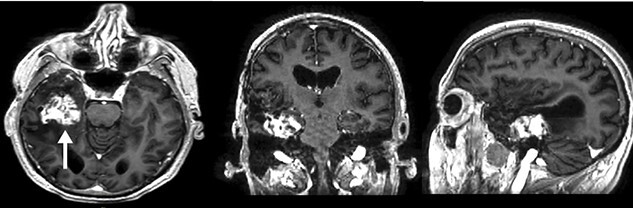
: (A–C) A surveillance contrast T1W MRI (axial, coronal and sagittal) conducted at 33 months demonstrating lesional recurrence in the right temporal lobe (arrow) and minimal mass effect prior to re-operation.

**Figure 5:**
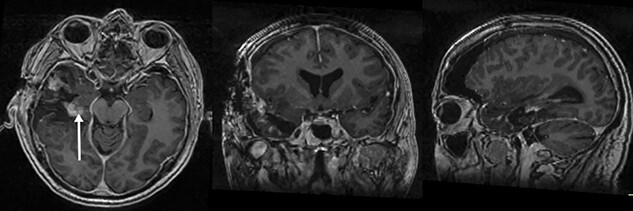
: (**A**–**C**) Post-operative contrast T1W MRI (axial, coronal and sagittal) showing significant lesional reduction but medial lesional enhancement (arrow) representing a STR.

**Figure 6:**
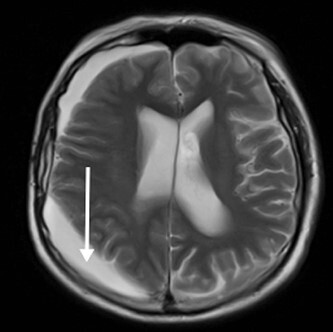
T2W MRI (axial) conducted at 36 months from original presentation demonstrating a right sided subdural collection consistent radiologically to a hygroma (arrow).

A surveillance MRI in February 2017 (Fig. 4**A**–**C**) demonstrated 3.5 × 3.0 × 2.0 mm (24 720 mm^3^) medial recurrence. The patient underwent re-resection and a 93% STR was achieved (Fig. 5**A**–**C**). The post-operative histology and molecular markers remained consistent. Palliative chemotherapy with TMZ was commenced. Following his third cycle, he was readmitted in May 2017 due to a week’s history of headache and transient facial weakness. MRI showed a sizeable right-sided subdural hygroma (Fig. 6).

As his symptoms spontaneously resolved and there was no neurological deficit, this was managed conservatively. TMZ was continued and repeat imaging in August demonstrated improved appearances. An MRI in December 2017 showed new contrast enhancing areas in the resection cavity indicative of disease recurrence. He was switched to procarbazine and lomustine for 6 weeks. In March 2018 following his second cycle, and his KPS deteriorating to < 50, his treatment was stopped and supportive care instituted as a strategy due to further disease progression on MRI and worsening side effects of chemotherapy. The patient died on May 2018: 3 years and 11 months from initial diagnosis.

## DISCUSSION

The incidence of glioblastoma is rising [2]. About 3–5% of long-term survivors (> 24 months) have a mean age of ~ 37 years. This is rare in advancing age [3]. Almost all studies demonstrate a negative correlation between increasing age and survival [3]. Patients > 65 years are considered higher risk for poorer outcomes due to poorer physiological reserve, therapy resistance and neurocognitive function [4] irrespective of other prognostic factors, e.g. molecular markers [5]. However, outliers do exist [6]. Consequently, long-term survival is probably multifactorial.

Our patient had an STR. A gross total resection (no demonstrable contrast-enhancing residual tumour) is a positive prognostic marker in overall survival (OS) [3]. Furthermore, OS is strongly dependent on adjuvant chemotherapy. The decision to offer such treatment is dependent on patient, imaging and surgical characteristics [4, 5]. A poor performance status (PS) is an independent risk factor for mortality 12-month following treatment [4, 6]. Our patient had a good PS until palliation. Therefore, assessment of patients should not centre on identifying advantageous molecular targets in isolation to broader clinical parameters. Scoring systems calculating co-morbidity and predicting mortality do exist, e.g. Cumulative Illness Rating Scale and Charlson Comorbidity Index [7]. Regardless of age, it is imperative to identify patients who have an actual capacity to benefit from treatment.

The landmark Stupp protocol established the standard of care [8]. It ironically excluded patients > 70 years. Recent studies demonstrate that O^6^-methylguanine-DNA methyltransferase (MGMT) methylation combined to TMZ and short-course XRT improved survival compared with short-course XRT in older patients (suggesting aggressive treatment can be tolerated) [2]. Our patient had MGMT methylation. MGMT encoded DNA-repair proteins remove alkylating agents causing chemotherapy resistance. Methylation results in transcriptional silencing increasing OS from TMZ and XRT [6]. MGMT methylated GBMs are localized more to frontal lobes conferring a better prognostic outlook [9]. Ironically, our patient had a temporal tumour. It is hypothesized that LEV in conjunction to TMZ could result in increased OS compared with TMZ alone [10] It enhances MGMT silencing. Our patient was on LEV.

Overall, a single biomarker is unlikely to correlate to a meaningful clinical response in all patients. The complex molecular cascade requiring activation to enable an anti-neoplastic response coupled to the molecular complexity of glioblastoma dictates this. Clearly, our patient demonstrated advantageous biology to endure two craniotomies and adjuvant oncology treatments. This highlights a need for individualized care.

## CONCLUSION

We demonstrate that older age does not exclude long-term survival of glioblastoma. With an aging population, it is ever more critical to individualize patient care in a holistic fashion.

## CONFLICTING OF INTEREST

No conflicts of interest.

## FUNDING

This research received no specific grant from any funding agency in the public, commercial or not-for-profit sectors.

## ETHICAL APPROVAL

This has patient consent. But no ethical approval was needed.

## CONSENT

Written informed consent has been obtained from the patient.

## GUARANTOR

Simon Lammy is the guarantor of this manuscript.
